# The Meharry Cancer Summer Undergraduate Research Program: A Strategy to Increase Undergraduate Exposure to Cancer Health Disparities

**DOI:** 10.1007/s13187-025-02762-4

**Published:** 2025-10-29

**Authors:** Evangeline Motley-Johnson, Margaret Whalen, LaMonica V. Stewart

**Affiliations:** 1 Department of Microbiology, Immunology and Physiology, Meharry Medical College, Nashville, TN 37208 USA; 2 Department of Chemistry, Tennessee State University, Nashville, TN 32709 USA; 3 Department of Biochemistry, Cancer Biology, Neuroscience and Pharmacology, Meharry Medical College, Nashville, TN 37208 USA

**Keywords:** Summer research program, Cancer health disparities, Cancer research education

## Abstract

A lack of diversity in the biomedical research workforce significantly affects our ability to address the public health burden and financial costs associated with cancer health disparities. To address this issue, we created the Meharry Cancer Summer Undergraduate Research Program (SURP). The Meharry Cancer SURP is a 10-week intensive research experience designed to expose undergraduate students to the area of cancer health disparities; provide students with a greater understanding of basic, clinical and translational cancer research; and to inspire participants to pursue cancer-focused careers. During the first six years of the program a nationwide search was performed to identify and select 10–17 participants per year. Students performed research projects with experienced investigators at either Meharry Medical College or Vanderbilt-Ingram Cancer Center. Each student participated in a week-long cancer biology mini course to ensure students understood fundamental concepts common to all cancers and relevance to the study of health disparities. They also attended research seminars given by oncologists and cancer researchers and career development sessions designed to provide information on cancer-focused career options and the tools required to submit competitive applications to medical and/or PhD graduate schools. Together these activities increased student awareness of cancer research and cancer health disparities. They also provided opportunities for participants to become part of the larger cancer research community.

## Introduction

Cancer health disparities are defined by the National Cancer Institute as “adverse differences between certain population groups in cancer measures, such as incidence, prevalence, morbidity, mortality, survivorship and quality of life after cancer treatment, burden of cancer or related health conditions, screening rates, and stage at diagnosis” [1]. Population groups that are affected by cancer health disparities include racial/ethnic minorities as well as people of low socioeconomic status. To reduce the financial and public health burden associated with cancer for all populations, we must identify strategies to reduce and/or eliminate these disparities. The American Society for Clinical Oncology (ASCO) has released two policy statements that contain recommendations for the reduction of cancer health disparities and promotion of health equity [2, 3]. One recommendation included in both statements was the need to increase the diversity of the cancer workforce. Members of the organization noted that this diverse workforce should include physicians and biomedical scientists who have training in clinical and biomedical research, for basic, clinical, translational and community-based research are required to study the complex problems that underlie cancer health disparities and identify effective strategies to solve these problems [2]. Furthermore, they mentioned the need to create environments that promote the development and maintenance of a diverse cancer workforce to address systemic barriers to health equity [3].

One factor that affects our ability to achieve a diverse cancer workforce is that few underrepresented minorities (URM) pursue careers as physicians and biomedical researchers. According to the Association of American Medical Colleges (AAMC), active physicians who identify as Black or African American, American Indian or Alaska Native, Native Hawaiian or other Pacific Islander, and/or Hispanic or Latino constitute approximately 13.4% of the physician workforce [4]. Furthermore, racial and ethnic minorities represent less than 10% of all female and less than 7% of all male medical school faculty [5]. This is due in part to the fact that fewer URMs hold M.D. and Ph.D. degrees. Few historically black colleges and universities (HBCUs) and other undergraduate institutions offer courses in cancer biology, which reduces student awareness of the field of cancer biology and cancer-focused careers. To increase the diversity of the cancer research workforce, we must encourage more URM students to pursue careers in science, medicine and cancer and provide them with the tools necessary to earn degrees that will help them be successful in these careers.

We created the Meharry Cancer Summer Undergraduate Research Program (SURP) to expose undergraduate students, especially those who are underrepresented in health-related sciences, to career options in cancer research. The primary objective of this program was to provide a 10-week intensive, hands-on paid research experience for undergraduate students (rising sophomores to rising seniors). This research experience was designed to increase student exposure to clinical, community-based and biomedical cancer research and increase student awareness of cancer health disparities. In addition, the program provided students with the information and tools needed to apply to M.D. and/or Ph.D. training programs to obtain the degrees needed for cancer-related careers. The goal of this paper is to outline the implementation strategy and program outcomes to date.

## Methods

### Recruitment and Selection of Program Participants

The Program Directors sent out a call for applications informing the undergraduate community about the Meharry Cancer SURP during November of each program year. A program brochure and a link to the online application were posted on the Meharry Medical College website, the Meharry School of Graduate Studies (SOGS) Facebook and Twitter pages, the Institute for Broadening Participation (IBP) Pathways to Science website, and other social media outlets. Brochures were also sent directly to faculty at our HBCU partner institutions (Clark Atlanta University, Fisk University, Tennessee State University, and Trinity Washington University). In addition, program directors advertised the program at the Annual Biomedical Research Conference for Minoritized Students (ABRCMS). The program application consisted of an online form, applicant transcripts and two letters of recommendation from faculty members or advisors. The annual deadline for submission of the application form and supporting documentation was February 5th.

An Admissions and Selection Committee was responsible for selecting the undergraduate participants each year. At least two Committee members reviewed each complete application. Each applicant was ranked on their academic performance (GPA), letters of recommendation, and statements within the online application form on 1) their future career goals and 2) interest in cancer research. A rubric was then used to award points 0–5 to the different categories with 5 being the highest. The scores were tallied, and applicants were ranked from those receiving highest to lowest scores. Rankings and additional written feedback from the Committee members regarding whether an applicant should be admitted were used to place applicants in one of three categories: 1) acceptance, 2) alternate, or 3) not to be invited. Applicant scores and categories were reviewed by the program directors before offers of acceptance were extended to applicants.

## Training Program Components

A. Research Training: A primary goal of the Meharry Cancer SURP was to increase undergraduate student exposure to clinical, community-based and biomedical cancer research and increase student awareness of cancer health disparities. All program participants during the summer of 2018–2019 and 2021–2023 completed an in-person cancer-focused research project. During the application process participants were asked to select their preference for research mentor and type of research project (basic science/translational, community-based research, or cancer epidemiology focused research). Program research mentors were selected based on their involvement in cancer/cancer health disparity research, their willingness to train undergraduate students, prior history of training undergraduates and/or other students, and current research funding. Our selected pool of mentors included basic/translational scientists and cancer epidemiologists/community outreach investigators that study cancers with a known cancer health disparity and/or perform research to examine disparities in cancer incidence, risk factors, screening, survival and patient education. Each applicant indicated their top three mentor choices from a list embedded within the online application that contained mentor names, research areas, and recent publications. Once applicants agreed to participate in the program, their list of top choices was used by program directors to pair participants with research mentors. Mentors then confirmed acceptance of their assigned summer trainee and received grant funds to support participant research training ($1,000 per student). Participants presented short oral talks and posters describing the results of their 10-week research project at a Summer Research Symposium held during the last week of the program. They were also given the opportunity to present their research at one national meeting. Participants were required to participate in sessions that outlined how to create and present research posters and short oral talks and write abstracts in preparation for these meetings.

Program participants also learned about different types of cancer research and cancer health disparities via cancer research seminars. At these seminars, established researchers in the fields of cancer biology and cancer health disparities discussed their approach to research projects and data obtained by their laboratories. They also provided the student participants with information about their career path and training. Speakers include oncologists, basic scientists, epidemiologists, and people who perform community-based research studies so that students obtain a broad view of the career options that are associated with cancer health disparities research. The seminar topics included breast cancer, prostate cancer, melanoma, cancer immunology, cancer bioinformatics, structural biology and cancer, cancer genetics and epidemiology.

B. Cancer Biology Mini-Course: During the first weeks of the program, students participated in a mini-course on cancer biology. The purpose of this course was to provide participants with an opportunity to apply basic concepts in biology, chemistry and biochemistry they had learned in college to the study of cancer. The course consisted of lectures given by Meharry faculty and Ph.D. graduate students that cover basic concepts in cancer biology common to all cancers. Course sessions included a discussion of methodologies commonly used in preclinical, clinical and community based/epidemiological research. To obtain a broad overview of the history of cancer, the students viewed and discussed episodes of the PBS documentary “Cancer: The Emperor of All Maladies”. An outline of the course schedule and lecture topics is shown in Table 1.

**Table 1:** Outline of cancer biology mini-course schedule

WEEKS 1 AND 2					
DAY 1	DAY 2	DAY 3	DAY 4	DAY 5	DAY 6
• Overview of Cancer • Introduction to Cancer Health Disparities	• Cancer Epidemiology • Cancer Related Signaling Pathways • Oncogenes and Tumor Suppressors • Discuss Emperor of All Maladies – Episode 1	• Causes of Cancer • Community Engaged Research	• The Scientific Process: How Do We Study Cancer? • Discuss Emperor of All Maladies – Episode 2	• Tumor Invasion and Metastasis • Cancer Chemotherapy Drugs • Cell Culture and Animal Models of Cancer	• Cancer-Related Societies • Discuss Emperor of All Maladies – Episode 3

C. Career Development Sessions: Participants attended weekly Career Development Seminars designed to help them develop the professional skills needed for success in biomedical sciences. Each summer program participants completed at least six career development sessions. Sessions were led by Meharry’s Office of Professional Development within the SOGS and SURP program faculty. The first session, which was entitled “Career Exploration”, focused on the different career options available to persons with the M.D., Ph.D. and M.D./Ph.D. degrees as well as strategies to define the best career options for each participant. Session two discussed cover letters and curriculum vitae/resumes. Students used these skills to submit applications for student membership to the American Association for Cancer Research. In session 3 participants learned how to prepare individual Development Plans (IDPs). The chairs of Meharry’s School of Medicine and School of Graduate Studies Ph.D. Admissions Committee along with the Assistant Director of the Medical Scientist Training Program (MSTP) at Vanderbilt University School of Medicine led session four, which was entitled “Admission to Medical and Graduate School”. During this session, students reviewed the requirements for entrance into medical school, Ph.D. graduate programs, and combined M.D./Ph.D. program at Meharry and Vanderbilt. During this session, panelists also shared the attributes of a successful interviewee. In a separate panel discussion, participants met with current Meharry M.D., Ph.D. and M.D./Ph.D. students and had the opportunity to ask questions about each student’s journey to professional school, daily activities and challenges. During session five, participants learned how to write personal statements. Each participant was asked to write a personal statement that was reviewed by the program directors. The sixth session was entitled “Interview Skills”. Attire, verbal and nonverbal communication, and a list of commonly asked questions were among the topics discussed with program participants. Participants also had an opportunity to participate in a Mock Interview session. Two additional sessions were added during the last year of the program: a session on informational interviews and a session on personality assessments. In these sessions, participants learned how to conduct focused interviews to gain information about career opportunities. They also used the PACE Pallet personality test to define their personality type and improve their communication skills.

D. Responsible Conduct of Research (RCR) Training: RCR was taught throughout the ten weeks of the summer program. The objective of this training was to introduce students to the ethical issues inherent to the conduct and dissemination of scientific research and to provide them with a framework in which to approach resolving the dilemmas that can arise. Six to seven 1-h RCR sessions occurred each summer. The sessions consisted of a 15–20-min didactic lecture followed by small group discussions. In breakout groups consisting of 3–4 participants/group, program participants discussed case studies involving different issues of scientific ethics and that covered relevant current and historical events. The topics covered for each RCR session were 1) Mentor/Mentee Relationships; 2) Introduction to moral reasoning and scientific misconduct; 3) Rigor and reproducibility/Study design; 4) Conflicts of interest and competing interests, 5) Data management, lab notebooks, recordkeeping and intellectual property; 6) Animal and human subjects; and 7) Authorship and publications.

## Program Evaluation

At the end of the program, participants were asked to complete two REDCap-based evaluation forms. The first evaluation asked questions about their experience with research mentors and within their respective research environments. Information gained from this evaluation was used to assess mentor effectiveness and inform student placement in subsequent program years. The second evaluation asked participants to comment on the impact of the program overall, their exposure to cancer health disparities, and various program components, including their thoughts on the cancer biology mini course, career development, RCR and seminar sessions. Research mentors also completed evaluation forms that provided feedback on each participant’s interest in research, understanding of research concepts, and research skills. This information was used to complete subsequent letters of recommendation for program participants.

### Results – Program Outcomes

## Recruitment Outcomes

The nation-wide recruitment strategy designed for this program was effective, for we received between 61–156 complete applications each year from interested undergraduate students (See Table 2). Applications were received from students who attended undergraduate institutions within the continental United States and Puerto Rico. From this applicant pool, we were able to recruit 10—17 program participants per year of the program. Each year the program cohort contained many women and URM students, with over 65% of the cohorts being female and at least 75% URM participants (Table 2).

**Table 2:** Demographics of Meharry Cancer SURP participants

YEAR	2018	2019	2020	2021	2022	2023
Number of Complete Applications	61	122	146	156	94	113
Total Number Programs Participants	10	10	13	10	12	17
Gender						
Female	8	9	9	7	10	14
Male	2	1	4	3	2	3
Race/Ethnicity						
Black or African-American	9	9	11	9	8	14
White	1	1	1	0	1	3
Native Hawaiian or Other Pacific Islander	0	0	1	0	0	0
American Indian/Alaska Native	0	0	0	1	1	0
Hispanic/Latino	0	0	0	0	0	0
Unknown/Not Reported	0	0	0	0	2	0

## Research Training and Presentation Outcomes

A total of 72 undergraduate students participated in the Meharry Cancer SURP since the program’s inception. Fifty-nine (59) students had the opportunity to complete in-person research projects. Of these, forty-nine (49) students performed basic and/or translational cancer research projects. The projects included studies that explored 1) signaling pathways and therapeutic responses in early and advanced human prostate cancer cells, 2) the biology of triple negative breast cancers (TNBC) and other breast cancer types, 3) the biological basis of health disparities in head and neck cancers, 5) differences in gene expression within colon cancer patients of different racial/ethnic backgrounds, and 6) the effect of obesity on TNBC and ovarian cancers. The remaining ten (10) students completed projects that involved cancer epidemiology and/or community engagement. These projects included studies that examined 1) the effect of dietary exposures on prostate cancer risk in different populations, 2) prostate cancer screening rates among different populations, and 3) differences in breast cancer risk factors across populations. In summer 2023, additional funding via a NIH supplement grant allowed us to provide training in cancer bioinformatics. As a result, five (5) program participants completed projects that included analysis of publicly available datasets as a key component of their research study. The types of cancers studied as part of the participant research projects were cancers known to be associated with cancer health disparities, including breast, prostate, colon and ovarian cancer.

During summer 2020, restrictions associated with the COVID-19 pandemic prevented in-person research projects. To ensure students had an opportunity to receive training in cancer health disparities, the summer 2020 program was converted to a virtual format. This 6-week virtual version of the program included all program components that did not require hands-on research. Sessions were hosted via Blackboard Collaborate or Zoom, and included the cancer biology mini course, career development sessions, RCR and cancer research seminars described above.

All program participants who completed research projects presented their research locally at Meharry during the Summer Research Symposium. Most program participants (57 out of 59 students) also presented posters describing their research projects at a national meeting. Of the students who attended national meetings, five (5) students presented their work at the 2019 AACR Undergraduate Student Caucus and Poster Competition, while one (1) student presented a poster at the Experimental Biology 2019 Annual Meeting. The remaining 51 students (86.4%) presented their research at ABRCMS. Four students won awards for their ABRCMS poster presentations (three in 2021, and one in 2022). This recognition reflects the quality of the research projects, and the presentation skills participants learned as part of the program.

## Summary Evaluation Feedback

**Fig. 1:**
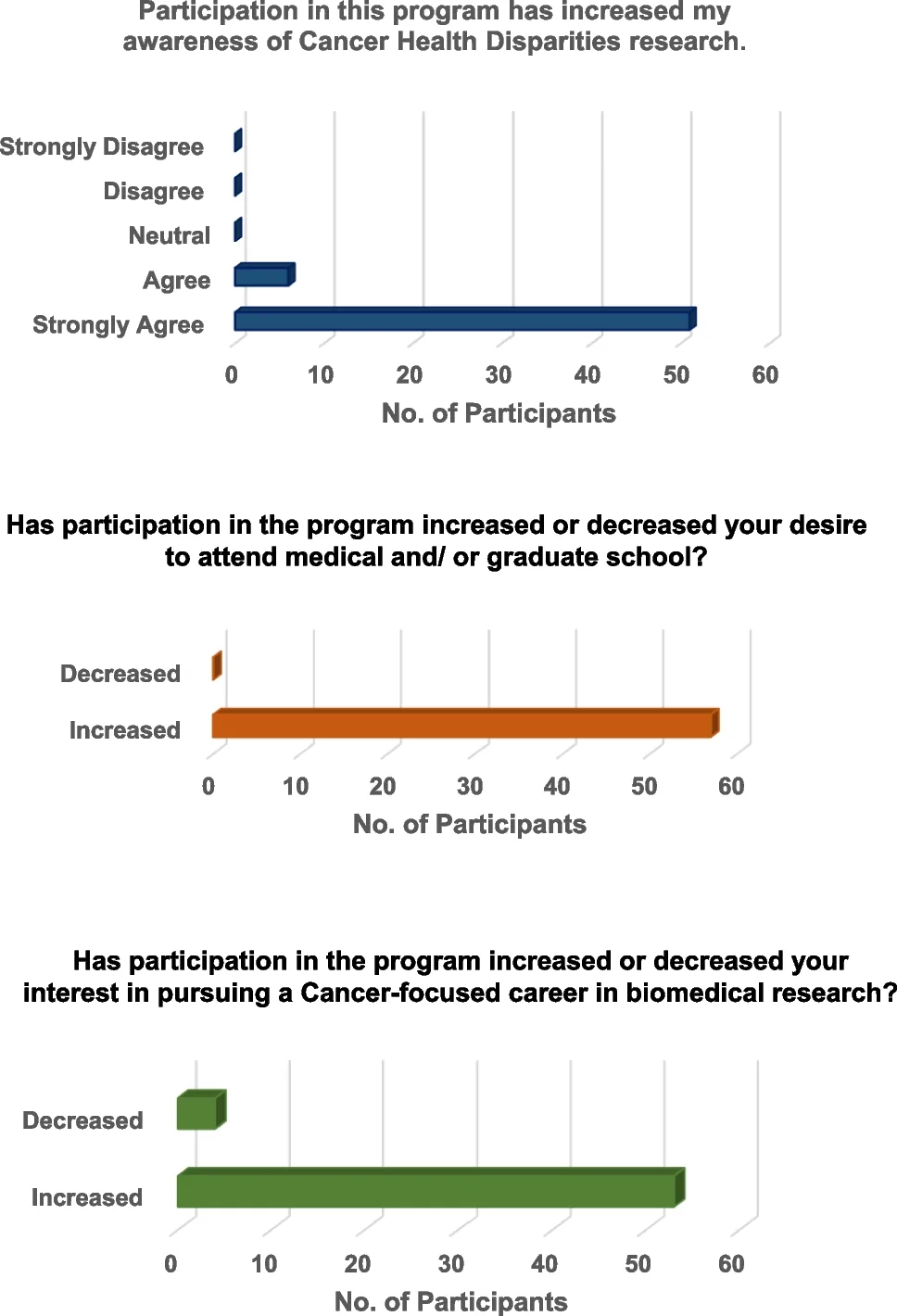
Summary results for Meharry Cancer SURP program-end evaluations

Summary data from program-end evaluation surveys suggest that the Meharry Cancer SURP was a great platform for students to learn about the area of cancer health disparities. All participants that completed research projects noted that the program increased their awareness of cancer health disparities. They also felt that the program increased their desire to attend medical and/or graduate school (Fig. 1). In addition, most of these participants (53 of 57, or 93%) felt this research experience increased their desire to pursue a cancer-focused research career. While summer 2020 participants did not have the opportunity to complete research projects, they felt the virtual version of the program increased their knowledge of cancer health disparities. We plan to perform additional follow-up surveys to determine the long-term impact of the program and the extent to which participants pursued cancer-focused careers in health disparities research and/or medicine.

## Conclusion

Since its establishment in 2017, the Meharry Cancer SURP has served as an effective platform to expose undergraduate students to the area of cancer health disparities. Undergraduate students who participated in the program learned foundational concepts of cancer biology and cancer health disparities and how to apply those concepts in hands-on research experiences, a cancer biology mini-course and research seminars. Participants became active members of the cancer research community and were able to share their research findings at national meetings. Program participants also interacted with faculty, staff and students who have served as role models and examples of people who have successful cancer-focused research careers. They took advantage of opportunities for near-peer mentoring with Meharry medical and PhD graduate students in their research environments and during program sessions. The program’s success is reflected not only in the number of national poster presentations but also by the fact that many participants have completed their undergraduate degrees and transitioned to medical school and/or science-focused graduate or health professional programs at Meharry and other institutions. In the next phase of the program, we plan to increase the types of cancer research experiences available to participants and expand training opportunities to first-year medical students. We will also use pre/post-session surveys to better assess student knowledge of cancer health disparities concepts. We believe these modifications will enhance our ability to increase student exposure to cancer research and address the nation’s biomedical research workforce needs.
